# Prognostic significance of LncRNA GHET1 expression in various cancers: a systematic review and meta-analysis

**DOI:** 10.1042/BSR20190608

**Published:** 2019-10-11

**Authors:** Jing Ye, Haiyan Sun, Zhengquan Feng, Qiqin Zhang, Yongliang Xia, Yunxi Ji, Qiqing Zhang

**Affiliations:** 1 Department of Medical Oncology, Tongde Hospital of Zhejiang Province, Hangzhou 310012, China; 2 Department of Traditional Chinese Internal Medicine, The First Affiliated Hospital of Zhejiang Chinese Medical University, Zhejiang Provincial Hospital of TCM, Hangzhou 310006, China; 3 Medical Insurance Management Office, The First Affiliated Hospital of Zhejiang Chinese Medical University, Zhejiang Provincial Hospital of TCM, Hangzhou 310006, China

**Keywords:** Cancer, LncRNA GHET1, Meta-analysis, Prognosis

## Abstract

*Background:* Dysregulated expression of long non-coding RNA gastric carcinoma high expressed transcript 1 (lncRNA GHET1) has been observed in several cancers, however, definite conclusion on the prognostic value of lncRNA GHET1 expression in human cancers has not been determined. The aim of this meta-analysis was to evaluate the prognostic significance of lncRNA GHET1 expression in cancers. *Methods:* PubMed, Web of Science and Embase were comprehensively searched for relevant studies. Meta-analyses of overall survival (OS) and clinicopathological features were conducted. *Results:* Ten studies were finally analyzed in the present study. High lncRNA GHET1 expression was associated with shorter OS than low lncRNA GHET1 expression in cancers (hazard ratio (HR) = 2.59, 95% CI = 1.93–3.47, *P*<0.01). Online cross-validation using The Cancer Genome Atlas (TCGA) data observed similar results (HR = 1.10, *P*<0.05). When compared with low lncRNA GHET1 expression, high lncRNA GHET1 expression was related to larger tumor size (*P*<0.01), worse differentiation (*P*<0.01), earlier distant metastasis (*P*=0.02), earlier lymph node metastasis (*P*<0.01) and more advanced clinical stage (*P*<0.01). *Conclusion*: High lncRNA GHET1 expression is associated with worse cancer prognosis and can serve as a promising prognostic factor of human cancers.

## Background

Cancer has become a leading cause of death and a vital public health problem worldwide [1,2]. Although great advancements have been achieved in the diagnosis and treatment of cancers in recent years, many people suffer from disappointing results [2]. The lack of efficient biomarkers to supervise the clinical outcomes and predict the prognosis is supposed to be an important reason for the poor prognosis of cancer patients [3–6].

Long non-coding RNA (lncRNA), longer than 200 nucleotides, is an important member of non-coding RNA family [7]. A great number of studies have found that lncRNAs play a crucial role in the development of human diseases although lncRNA is short of the ability to code proteins [7,8]. Recently, accumulating evidence shows lncRNA is involved with tumor tumorigenesis, invasion and metastasis [9]. Several lncRNAs have been identified as prognostic factors in cancers, such as metastasis-associated lung adenocarcinoma transcript 1 (MAlAT1) [10] and cancer susceptibility 2 (CASC2) [5]. Gastric carcinoma high expressed transcript 1 (GHET1), a kind of lncRNA with the length of 1913 nt, is located at chromosome 7q36.1 position in the human genome [11]. Recently, many studies found that lncRNA GHET1 contributed to the cancer progression and had the potential ability to predict the cancer prognosis [12–21]. However, definite conclusion has not been obtained for contradictory results among different studies. For instance, Xia et al. [19] study showed there was no obvious relationship between lncRNA GHET1 expression and lymph node metastasis (*P*=0.41), similar results were observed in Yang et al. study (*P*=0.20) [20]. Nevertheless, Liu et al. [15] detected the significant association between lncRNA GHET1 expression and lymph node metastasis (*P*<0.01), similarly, Shen et al. [17] also discovered the evident connection between high lncRNA GHET1 expression and earlier lymph node metastasis (*P*<0.01). In view of these conflicting data, for the first time, we performed this systematic review and meta-analysis to evaluate the prognostic significance of lncRNA GHET1 expression in cancers.

## Materials and methods

### Literature search and selection

We searched PubMed, Web of Science and Embase using the following strategy: (‘long non-coding RNA’ OR ‘lncRNA’) AND (‘gastric carcinoma high expressed transcript 1’ OR ‘GHET1’) AND (‘tumor’ OR ‘cancer’ OR ‘carcinoma’). The last literature search was conducted on 5 May 2019. Literature selection was performed according to inclusion and exclusion standards. Two authors completed the literature search and selection independently, and any disagreement was solved by group discussion.

### Inclusion standards and exclusion standards

The study would be included into this research if it met the following inclusion standards: (i) patients were diagnosed with cancers; (ii) patients with high lncRNA GHET1 expression were divided into research group; (iii) patients with low lncRNA GHET1 expression were divided into control group; (iv) association of lncRNA GHET1 expression with overall survival (OS), progression-free survival (PFS) or clinicopathological parameters was reported; (v) studies contained retrospective cohorts or perspective cohorts. The following studies were directly excluded from this meta-analysis: reviews, comments, letters, animal experiments, cell experiments, duplicated publications or studies without sufficient data.

### Data extraction and quality assessment

We extracted the following items using a prepared template: first author, publication year, country, sample size, gender, lncRNA GHET1 expression, cut-off value, detection methods, type of cancer and outcomes. Especially, hazard ratio (HR) and 95% confidence interval (CI) of OS were directly obtained from published studies. If HR and corresponding 95% CI were not directly reported, both of them could be indirectly extracted from survival curves used Engauge Digitizer 4.1 [22]. Quality of each included study was assessed using Newcastle–Ottawa Scale (NOS) [23]. The study with NOS ≥ 6 was considered as high-quality study. The process of data extraction and quality assessment was completed by two authors independently. Any disagreement was solved by group discussion.

### Online cross-validation

We conducted online cross-validation to validate the prognostic role of lncRNA GHET1 expression in human cancers using Gene Expression Profiling Interactive Analysis (GEPIA) (http://gepia.cancer-pku.cn/index.html) based on The Cancer Genome Atlas (TCGA) data (https://cancergenome.nih.gov/).

### Statistical analysis

HR and corresponding 95% CI were pooled to determine the association between lncRNA GHET1 expression and OS. Odds ratio (OR) and 95% CI were used to assess the relationship between lncRNA GHET1 expression and clinicopathological features, such as age, gender and tumor size. Heterogeneity was assessed via chi-square-based Q and *I^2^* tests across studies. A fixed-effect model was used when the heterogeneity was obvious (*I^2^* > 50 or *P*<0.05). Otherwise, a random-effect model was applied (*I^2^* ≤ 50 or *P*≥0.05). Forest plot was applied to show the overall effects. Funnel plot, Begg’s test and Egger’s test were generated to evaluate the publication bias. Sensitivity analysis was conducted to check the robustness of results by omitting one study at a time. All analyses were performed using Review Manager 5.3 (The Cochrane Collaboration, Copenhagen, Denmark) and Stata 12.0 (Stata, College Station, TX, U.S.A.). All *P*-values were two-sided and a *P*-value less than 0.05 indicated the results were statistically significant.

## Results

### Literature search and selection

**Figure 1:**
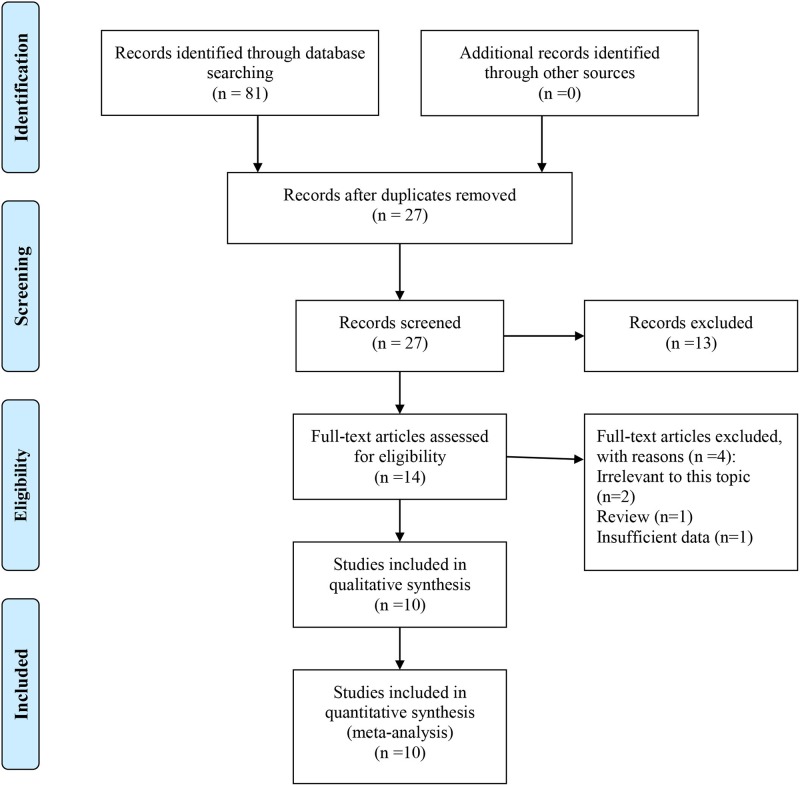
Literature search and selection

As shown in Figure 1, a total of 81 papers were retrieved from three common databases. After removal of duplicates, 27 papers remained for further evaluation. Then, 13 papers were directly excluded by scanning titles or abstracts. The remaining 14 papers were further checked for eligibility by reading full-texts, and then 4 papers were removed. Ultimately, ten studies were included into this systematic review and meta-analysis.

### Characteristics of included studies

Characteristics of included studies were listed in Table 1. A total of 654 patients (350 males and 304 females) were included in this research [12–21]. All studies were conducted in China and sample size varied from 42 to 105 [12–21]. There were 323 and 331 patients in high lncRNA GHET1 expression group and low lncRNA GHET1 expression group, respectively. The expression level of lncRNA GHET1 was evaluated using quantitative real-time polymerase chain reaction (qRT-PCR) in all studies [12–21]. Besides, nine studies used median value [12,14–21] and one study used mean value [13] as the cut-off value. Eight kinds of cancer were investigated, including non-small-cell lung carcinoma (NSCLC) [12,17], hepatocellular carcinoma (HCC) [13], bladder cancer [14], esophageal squamous cell carcinoma (ESCC) [16], head and neck cancer (HNC) [15], breast cancer [18], gastric cancer [19,20] and pancreatic cancer [21]. Moreover, all studies reported clinicopathological parameters [12–21], seven studies reported OS [12–15,17,18,20] and one study reported PFS [17]. NOS was equal to or greater than six in all studies, which suggested all studies were of high quality [12–21].

**Table 1:** Characteristics of included studies

Study	Country	Sample size (*n*)	Gender (M/F) (*n*)	GHET1 expression (H/L) (*n*)	Detection method	Cut-off value	Cancer	Outcomes	NOS
Guan (2017) [12]	China	52	40/12	25/27	qRT-PCR	Median	NSCLC	CP, OS	7
Jin (2017) [13]	China	68	35/33	27/41	qRT-PCR	Mean	HCC	CP, OS	7
Li (2014) [14]	China	80	43/37	39/41	qRT-PCR	Median	Bladder cancer	CP, OS	7
Liu (2017) [16]	China	55	34/21	28/27	qRT-PCR	Median	ESCC	CP	6
Liu (2018) [15]	China	86	61/25	43/43	qRT-PCR	Median	HNC	CP,OS	7
Shen (2018) [17]	China	105	44/61	53/52	qRT-PCR	Median	NSCLC	CP, OS, PFS	7
Song (2018) [18]	China	60	0/60	30/30	qRT-PCR	Median	Breast cancer	CP, OS	7
Xia (2018) [19]	China	42	28/14	21/21	qRT-PCR	Median	Gastric cancer	CP	6
Yang (2014) [20]	China	42	31/11	21/21	qRT-PCR	Median	Gastric cancer	CP, OS	7
Zhou (2017) [21]	China	64	34/30	36/28	qRT-PCR	Median	Pancreatic cancer	CP	6

Abbreviations: CP, clinicopathological parameter; F, female; H, high GHET1 expression; L, low GHET1 expression; M, male.

### Meta-analysis of OS

**Figure 2:**
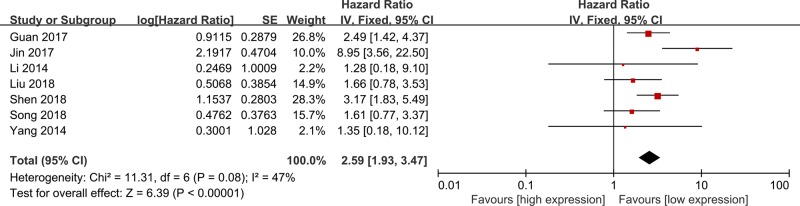
Meta-analysis of OS

Seven studies were included in the meta-analysis of OS (Figure 2) [12–15,17,18,20]. A fixed-effect model was used for mild heterogeneity across included studies (*I^2^* = 47%, *P*=0.08), and results showed high lncRNA GHET1 expression was significantly associated with shorter OS than low lncRNA GHET1 expression in cancers (HR = 2.59, 95% CI = 1.93–3.47, *P*<0.01). Subgroup analyses also indicated the obvious relationship between high lncRNA GHET1 expression and shorter OS in cancers (*P*<0.05) (Table 2).

**Table 2:** Subgroup analysis of OS

Variables	Studies (*n*)	HR, 95%CI	*P*	Heterogeneity		Model
				*I^2^* (%)	*P*	
**Sample size (*n*)**						
≤60	3	2.07 (1.34, 3.21)	<0.01*	0	0.60	Fixed
>60	4	3.10 (1.47, 6.55)	<0.01*	65	0.04	Random
**Cut-off value**						
Median	6	2.26 (1.66, 3.07)	<0.01*	0	0.61	Fixed
Mean	1	8.95 (3.56, 22.50)	<0.01*	NA	NA	Fixed
**Cancer type**						
NSCLC	2	2.82 (1.90, 4.18)	<0.01*	0	0.55	Fixed
Others	5	2.34 (1.06, 5.16)	0.03*	62	0.03	Random

Abbreviation: NA, not available. *P<0.05 indicating significant association between GHET1 expression and OS.

### Online cross-validation

**Figure 3:**
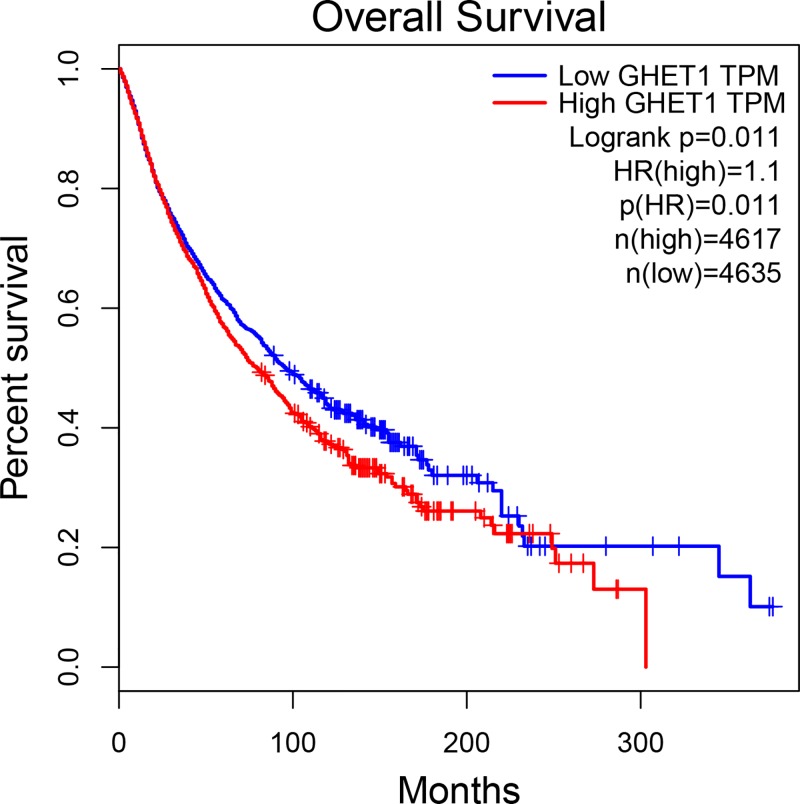
Online cross-validation using TCGA data

As shown in Figure 3, online cross-validation using TCGA data showed patients with high lncRNA GHET1 expression tended to have shorter OS compared with those with low lncRNA GHET1 expression (HR = 1.10, *P*<0.05).

### Meta-analysis of clinicopathological features

As listed in Table 3, there was no distinct relationship between lncRNA GHET1 expression and age (*P*=0.70) or gender (*P*=0.74). Nevertheless, high lncRNA GHET1 expression was obviously related to larger tumor size (*P*<0.01), worse differentiation (*P*<0.01), earlier distant metastasis (*P*=0.02), earlier lymph node metastasis (*P*<0.01) and more advanced clinical stage (*P*<0.01) than low lncRNA GHET1 expression in cancers.

**Table 3:** Association between GHET1 expression and clinicopathological features

Variables	Studies (*n*)	Patients (*n*)	High expression group (%)	Low expression group (%)	OR 95% CI	*P*	Heterogeneity		Model
							*I^2^* (%)	*P*	
Age (old versus young)	9	574	50.7 versus 49.3	52.1 versus 47.9	0.94 (0.67, 1.31)	0.70	0	0.88	Fixed
Gender (male versus female)	8	514	59.1 versus 40.9	60.4 versus 39.6	0.94 (0.65, 1.35)	0.74	0	0.81	Fixed
Tumor size (large versus small)	8	522	62.9 versus 37.1	35.7 versus 64.3	3.06 (2.14, 4.38)	<0.01*	45	0.08	Fixed
Tumor differentiation (poor versus well)	6	345	63.6 versus 36.4	44.4 versus 55.6	2.32 (1.48, 3.64)	<0.01*	27	0.23	Fixed
Distant metastasis (yes versus no)	3	148	15.4 versus 84.6	2.9 versus 97.1	4.63 (1.23, 17.38)	0.02*	0	0.59	Fixed
Lymph node metastasis (yes versus no)	7	442	59.7 versus 40.3	30.3 versus 69.7	3.81 (2.51, 5.77)	<0.01*	49	0.07	Fixed
Clinical stage (III/IV versus I/II)	6	422	62.8 versus 37.2	30.4 versus 69.6	3.92 (2.60, 5.91)	<0.01*	0	0.92	Fixe

*P<0.05 indicating significant association between GHET1 expression and clinicopathological features.

### Publication bias and sensitivity analysis

**Figure 4:**
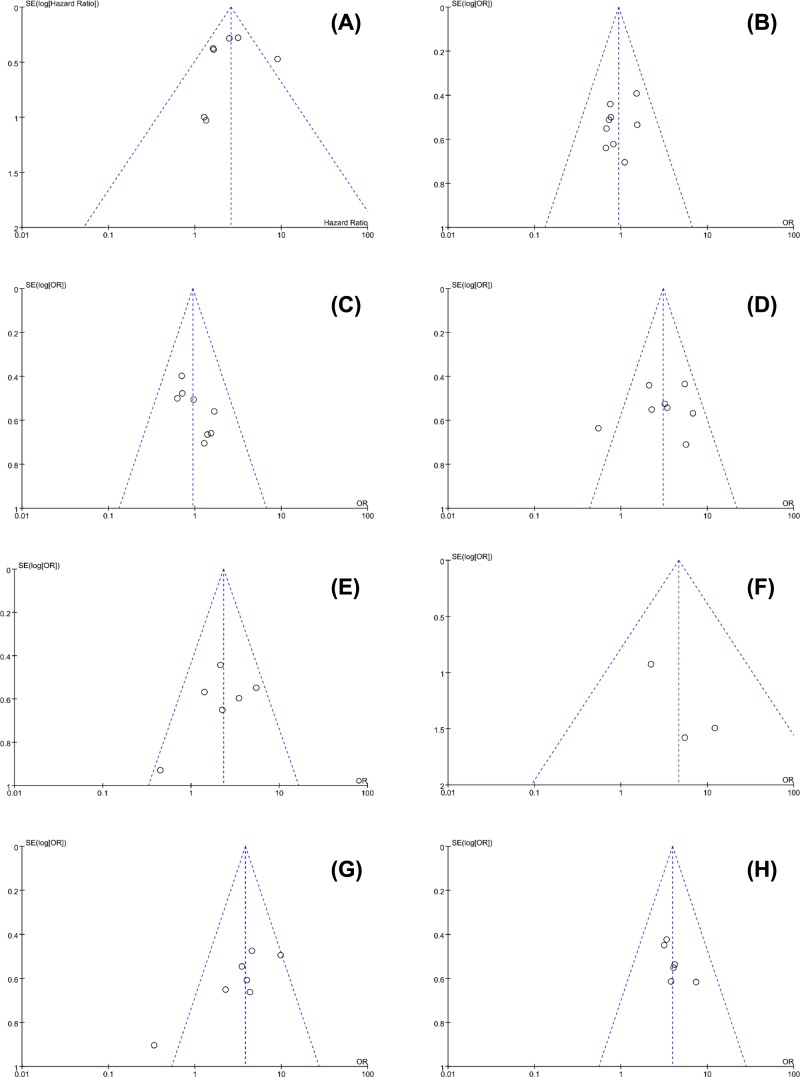
Funnel plots for all meta-analyses

**Figure 5:**
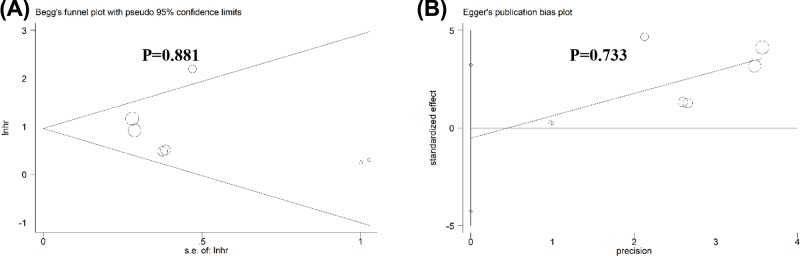
Begg’s test and Egger’s test for the meta-analysis of OS

**Figure 6:**
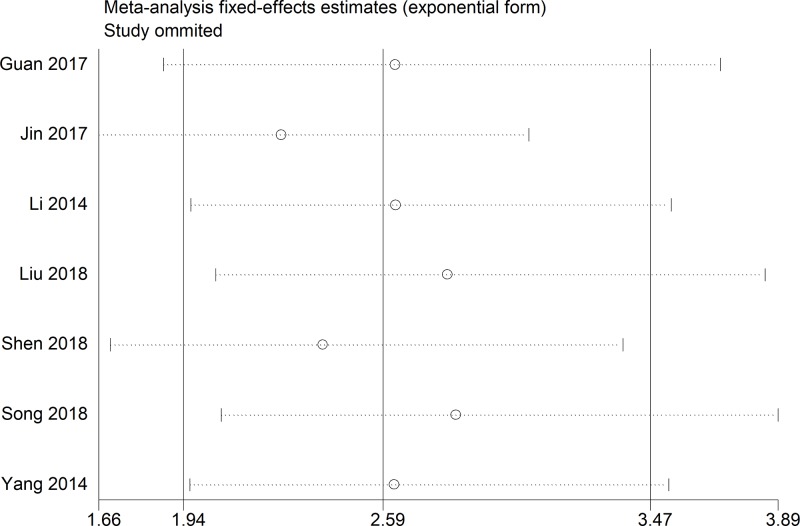
Sensitivity analysis for the meta-analysis of OS

No obvious publication bias across included studies was observed in all analyses (Figure 4). Especially, as for the meta-analysis of OS, publication bias was also assessed using Begg’s test (*P*=0.881) and Egger’s test (*P*=0.733), and no distinct publication bias was found (Figure 5). Sensitivity analysis for the meta-analysis of OS was conducted, and results were not altered after removal of any included study (Figure 6).

## Discussion

LncRNA has been proved to associate with cancer tumorigenesis, invasion, differentiation and metastasis [24]. Several lncRNAs have been demonstrated as prognostic biomarkers of human cancers [5,25]. Although accelerating evidence indicates lncRNA GHET1 may have the potential ability to predict the cancer prognosis, clear mechanism has not been obtained. Guan et al. [12] found that knockdown of lncRNA GHET1 could suppress the proliferation and invasion capacity of NSCLC cells by suppressing LATS1/YAP pathway signaling pathway in NSCLC cells. Xia et al. [19] discovered that down-regulation of lncRNA GHET1 inhibited the migration, invasion and proliferation of gastric cancer cells via up-regulating P21 expression and down-regulating cyclin and CDK expression to inhibit the G_0_/G_1_ to S phase transition. Song et al. [18] found lncRNA GHET1 promoted the cancer progression via EMT in breast cancer. Jin et al. [13] found lncRNA GHET1 facilitated the HCC cell proliferation by silencing KLF2 and further caused disappointing results. Ding et al. [26] study showed overexpression of ATF1 reversed the lncRNA GHET1 knockdown-mediated inhibition on the progression of HCC cells. Yang et al. [20] observed that lncRNA GHET1 promoted cancer cell proliferation by increasing c-Myc mRNA stability in gastric cancer.

In order to determine the prognostic significance of lncRNA GHET1 expression in human cancers, we performed this meta-analysis by integrating the current evidence [12–21]. To our knowledge, the present study was the first meta-analysis to evaluate the association between lncRNA GHET1 expression and cancer prognosis. We discovered that, compared with low lncRNA GHET1 expression, high lncRNA GHET1 expression was associated with worse OS and several clinicopathological features, including tumor size, differentiation, distant metastasis, lymph node metastasis and clinical stage. Besides, online-cross validation also indicated that high lncRNA GHET1 expression was an unfavorable prognostic factor of cancer. Overall, lncRNA GHET1 expression could serve as a potential prognostic biomarker for human cancers.

Some limitations should be considered when elucidating our data. First, only ten studies were included into this meta-analysis, and the relatively small sample size might lower the stringency of results. To eliminate this limitation, we have used TCGA data with a large population to validate our results, hence, we believe our study can provide reliable conclusion. Second, although we do not impose restrictions on the country when performing literature search and selection, all included studies are performed in China, which generates a region bias. However, as aforesaid, we have used TCGA data to validate the results, therefore, we believe our conclusion can be extended into other countries. Third, the cut-off value of lncRNA GHET1 expression varies a lot among different studies, as a result, definite cut-off value has not be obtained, which may limit the clinical use of our conclusion. Nevertheless, as aforesaid, this meta-analysis is a preliminary study to acknowledge the prognostic significance of lncRNA GHET1 expression in cancers, and more researches should be carried out to identify the optimal cut-off value of lncRNA GHET1 expression in future.

## Conclusion

High lncRNA GHET1 expression is associated with worse OS and clinicopathological features compared with low lncRNA GHET1 expression in human cancers. LncRNA GHET1 expression can serve as a promising prognostic factor of cancers.

## Abbreviations

CI: confidence interval
GHET1: gastric carcinoma high expressed transcript 1
HCC: hepatocellular carcinoma
HR: hazard ratio
LncRNA: long non-coding RNA
NOS: Newcastle–Ottawa scale
NSCLC: non-small-cell lung carcinoma
OS: overall survival
PFS: progression-free survival
qRT-PCR: quantitative real-time polymerase chain reaction
TCGA: The Cancer Genome Atlas
